# Prevalence of undiagnosed type 2 diabetes in patients admitted with acute coronary syndrome: the utility of easily reproducible screening methods

**DOI:** 10.1186/s12902-017-0153-y

**Published:** 2017-01-23

**Authors:** Muhammad A. Karamat, Umar Y. Raja, Susan E. Manley, Alan Jones, Wasim Hanif, Abd A. Tahrani

**Affiliations:** 1 Department of Endocrinology and Diabetes, Heartlands Hospital Birmingham, Birmingham, UK; 2 Department of Diabetes, Queen Elizabeth Hospital, Birmingham, UK; 3 Institute of Metabolism and Systems, School of Clinical and Experimental Medicine, University of Birmingham, Edgbaston, Birmingham, B15 2TT UK; 4 Department of Clinical Chemistry, Queen Elizabeth Hospital, Birmingham, UK; 5 Department of Clinical Chemistry, Heartlands Hospital Birmingham, Birmingham, UK; 6 Centre of Endocrinology, Diabetes and Metabolism, Birmingham Health Partners, Birmingham, UK

## Abstract

**Background:**

Despite the recognition of the importance of diagnosing dysglycaemia in patients with acute coronary syndrome (ACS) there remains a lack of consensus on the best screening modality. Our primary aims were to determine the prevalence of undiagnosed dysglycaemia and to compare the OGTT and HbA1c criteria for diagnosis of T2DM in patients admitted to hospital with ACS at baseline and at 3-months. We also aimed to investigate the role of a screening algorithm and a predictor score to define glucose tolerance in this population.

**Methods:**

A prospective study in which patients admitted with ACS to two UK teaching hospitals were assessed at baseline and 3 months follow-up.

**Results:**

The prevalence of diabetes at baseline was 20% and 16% based on OGTT and HbA1c criteria respectively. Forty three (43) % of the patients with T2DM based on OGTT would have been missed by the HbA1c criteria at baseline. Our screening algorithm identified 87% of patients with T2DM diagnosed with OGTT. Diabetes Predictor score had better sensitivity (>80%) and negative predictive value (>90%) compared to HbA1c criteria. Two thirds of participants with IGS and a third with T2DM changed their glycaemic status at 3 months.

**Conclusions:**

Only 48% of the patients admitted with ACS had normo-glycaemia based on OGTT. OGTT and HbA1c identified two different populations of patients with dysglycaemia with the HbA1c criteria missing almost half the patients with T2DM based on OGTT. Compared to HbA1c criteria our diabetes algorithm and diabetes predictor score had a better correlation with OGTT criteria.

## Background

Acute coronary syndrome (ACS) comprises a wide spectrum including non-ST segment elevation myocardial infarction (NSTEMI) and ST segment elevation MI (STEMI) [1, 2] and affects approximately 7 million people worldwide [2, 3]. Diabetes mellitus is a major risk factor for cardiovascular disease [4]. Dysglycaemia is associated with increased mortality and morbidity in patient with ACS as well as poor immediate outcomes [5–11]. Hospitalized patients with ACS have a high incidence of impaired glycaemic status (IGS) and Type 2 diabetes (T2DM) [10–15] with, 33% having impaired glucose tolerance (IGT) and 33% having T2DM [10]. Although the magnitude of this problem is increasingly being appreciated [10–15], the best mode for screening is unclear. Some reports indicate using an oral glucose tolerance test (OGTT) at the time of discharge from hospital in patients with ACS is a reliable method in predicting glycaemic status at 3 and 12 months [10]. However, these data also indicate an uncertainly of outcome since less than 50% of patients diagnosed with diabetes at the time of discharge still met the criteria at 12 months [10]. It is also clear from studies such as UKPDS and DCCT that early detection of diabetes prevents the development of long term complications [16–18]. 

The European Association for the study of Diabetes (EASD) recommends the use of an OGTT to investigate glycaemic abnormalities in patients with CVD without a known diagnosis of diabetes [19]. In contrast, this approach is not supported by the American Heart Association Diabetes Committee of the Council on Nutrition, Physical Activity and Metabolism [20]. This partly reflects the scarcity of conclusive evidence indicating that early intensive glycemic control improves cardiovascular outcomes. It is also unclear whether the acute dysglycaemia is a cause or effect of the myocardial ischemia and whether it justifies treatment per se or whether it should be viewed as a transient stress marker [21, 22]. Moreover, the complexity of performing an OGTT is also a factor: data from Holland suggest that 76% of cardiologists do not check an HbA1c in patients with ACS before discharge [23], making it unlikely that a more impractical test like OGTT would be used more frequently.

Until recently the diagnosis of diabetes was based on OGTT criteria [24]. However, the need for a simple and reliable screening tool has long been recognised and an International Expert Committee comprising the EASD, American Diabetes Association (ADA) and International Diabetes Federation (IDF) in 2009 recommended the use of an HbA1c cut-off of 6.5% (48 mmol/mol) for the diagnosis of diabetes provided the methodology was standardized and subjected to quality assurance protocols [25]. These criteria have now been adopted by the WHO.

We have developed a simple T2DM screening algorithm based on the FPG and HbA1c in the general public [26]. This was originally designed to limit the number of subjects requiring an OGTT in a group of patients referred due to abnormal impaired fasting glucose (IFG). The FPG identified 36% of patients with diabetes mellitus while OGTT identified a further 12%. The derived algorithm, [HbA1c ≥ 6.0% (42 mmol/mol) with FPG < 7.0 mmol/l] was utilized to identify patients requiring an OGTT to diagnose diabetes. When applied to the UK validation cohort, sensitivity was 97% and specificity 100%. Use of the algorithm would have reduced the number of OGTTs performed in the UK validation cohort by 33% and in the Australian cohort by 66%. Hence this could simplify procedures for diagnosis of diabetes, but validation was required in other patient groups [26]. Our primary aims were:

1. To determine prevalence of undiagnosed diabetes and impaired glycaemic state (according to OGTT criteria) in patients admitted with ACS.
2. To compare the OGTT and HbA1c criteria for diagnosis of T2DM in patients admitted to hospital with ACS at baseline and at 3-month follow up.
3. To investigate the role of simple and reproducible screening methods such as diabetes predictor score and screening algorithm to accurately define glucose tolerance in patients admitted with ACS.

## Methods

We conducted a cross-sectional and prospective study of adult patients who were admitted with ACS (STEMI and NSTEMI) to two secondary care inner-city hospitals in Birmingham UK between 2008 and 2010. One hundred and eighteen (118) Patients aged 18-90 years and not-known to have diabetes prior to admission were recruited. We excluded patients known to have diabetes (from past medical and medication history) or non-cardiac chest pain based on cardiac assessment. The patients were approached by a member of the research team (either a research nurse or a clinician) within 3 days of admission and baseline data and blood samples were obtained within 7 days of the cardiac event. The study was approved by the Birmingham, East, North and Solihull Research Ethics Committee (reference number 08/H1206/5) and all patients were consented as per the ethical approval.

### Assessments

Data collected during one-to-one interview and from electronic records included age, gender, ethnicity (determined in accordance with the UK decennial census by the study participants), medications, past medical history, family history, smoking history, history of hypertension and dyslipidaemia. Nature of the cardiac event, treatment received and outcome of underlying cardiac condition were also recorded. Other data included height, weight, body mass index (BMI), HbA1c, lipid profile, and blood pressure (BP). BP was measured by an automated Omron HEM-907 device (Omron Healthcare, Netherlands) whilst patient was seated with left arm resting horizontally. The average of 2 measurements taken at least 10 min apart after 30 min of rest was used.

### Blood sampling

Fasting plasma and serum samples were collected and were stored at -80 ^o^C following centrifugation. Glucose and HbA1c as well as basic biochemistry were carried out on fresh samples.

### Glucose

Glucose was measured using a hexokinase kit (Cat. No. 11876899216) and C.f.a.s. calibrator for automated systems (Cat. No. 10759350190) on a Roche Modular platform (Roche Diagnostics, E Sussex, UK) with coefficients of variance across the range of <2%.

### OGTT

OGTTs were performed at admission (within 7 days) and 3 months from baseline. Patients were requested to fast from the previous evening for 10 h and bloods collected the next morning. Plasma glucose and HbA1c were measured on venous blood. For the 75 g OGTT, patient was asked to drink 113 mL glucose polymer drink, Polycal, (Nutricia Clinical Care, Wiltshire, UK) over a period of 5 min. A further venous blood sample was taken after 2 h for plasma glucose measurement. Diagnosis of diabetes was based on OGTT criteria [24]. All patients received standard life-style advice given to post MI patients. Patients with HbA1c > 7.5% (58 mmol/mol) at baseline received glucose lowering treatment and did not have follow-up OGTT (n = 8/23, 35%).

### HbA1c

‘DCCT aligned’ HbA1c was reported from an ion exchange, high performance, liquid chromatography analyser, TOSOH G7 A1c Variant Mode (Tosoh Bioscience Ltd, Worcs, UK) that detects haemoglobin variants. A reference interval of <6% (42 mmol/mol) HbA1c was quoted by the manufacturer. HbA1c was not reported in patients with variant haemoglobins. There was one patient in our study identified with variant Hb.

### Screening algorithm

**Figure 1:**
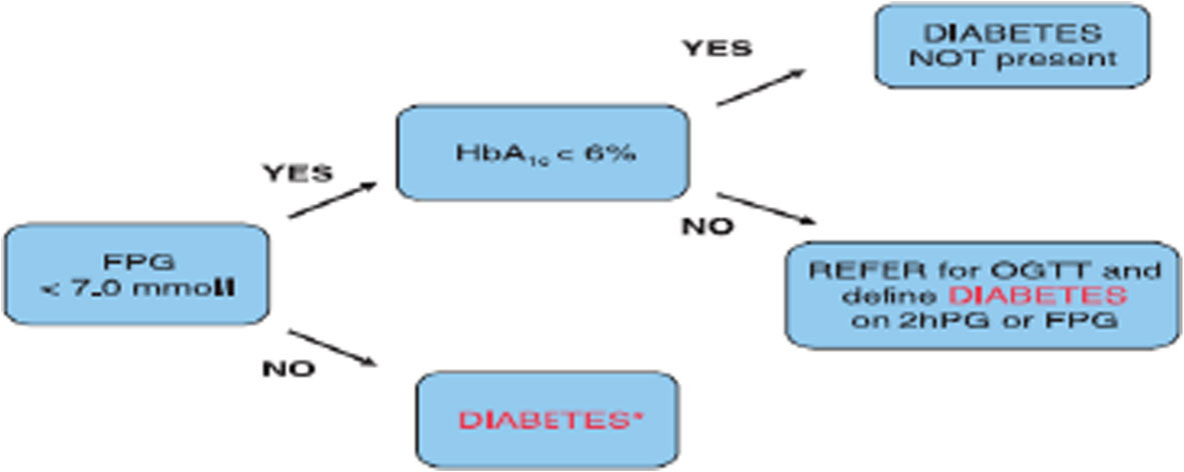
The screening algorithm

Our team has developed and published a T2DM screening algorithm based on FPG and HbA1c (Figure 1) [26]. It was derived from oral glucose tolerance test (OGTT) capillary samples in 500 consecutive UK patients referred with IFG and validated in 500 UK patients as well as venous specimens in 1175 unselected Australian patients [26].

### Statistical analysis

Based on previous publications [10] we expected a 33% prevalence of T2DM in our cohort. Using our screening algorithm [26] in order to achieve sensitivity of 97% (95% confidence interval 84-100%) and specificity of 94% (95% confidence interval 85-98%) we needed a sample size of 100 participants. Allowing for 10% drop outs we aimed to recruit 110 patients.

Data analyses were performed using SPSS 15.0 software (SPSS Inc, Chicago, IL). Data are presented as mean ± SD or frequencies. Independent continuous variables were compared using the Student *t* test. Categorical variables were compared using the Chi-square test. Correlations between continuous variables were performed using the Pearson or Spearman tests. Differences between independent groups were assessed by analysis of variance (ANOVA). If the homogeneity of variance assumption of ANOVA was violated, the Welch statistics were used to calculate the *P* values.

To assess the relationship between continuous and/or categorical variables and dichotomous outcomes multiple logistic regressions (forced entry method) was used. Variables included in the regression models were based on known outcome-related risk factors. We assessed multicolinearity in both multiple linear and logistic regression models using simple correlations between variables plus the tolerance values, and the condition indices. No tolerance values were < 0.1 and no variables had strong correlations (r > 0.8). In multiple linear regression models, the residuals were examined. In all the models presented, residuals followed a normal distribution with uniform variance and there was no relationship between the residual and predictor of interest. Data distribution was assessed using histograms and the Shapiro-Wilk test. A *p* value < 0.05 was considered significant unless stated otherwise.

## Results

One hundred and ninety eight (198) patients without diabetes were approached, of which 118 were consented. Participants’ clinical characteristics are summarised in Table 1. The study population was largely of middle age White men (81 male and 80% White Caucasian) (Table 1). Diagnoses of STEMI and NSTEMI were established in 43 and 57%, of the cohort respectively.

**Table 1:** Clinical and metabolic parameters of study participants

Variables	Mean	S.D	Median	IQR	Min	Max
Age (Years) (*n* = 118)	61	11.9	61	53-71	31	90
BMI (kg/m²) (*n* = 106)	28	5.1	28	25-32	17	47
Baseline FPG (mmol/L) (*n* = 118)	5.7	1.2	5.4	5.0-6.0	4.3	13.1
Baseline 2 hr PG (mmol/L) (*n* = 118)	8.5	3.7	7.7	5.9-9.9	2.4	23.5
Baseline HbA1c (%)^a^ mmol/mol (*n* = 117)	6.1 (43)	0.84	5.9	5.7-6.2	4.8 (29)	10.6 (92)
Follow up FPG (*n* = 92)^b^	5.7	0.99	5.6	5.2 – 6.1	2.1	10.4
Follow up 2 hr PG (*n* = 91)^b^	6.8	2.8	5.9	5-8.5	2.4	15.4
Follow up HbA1c (*n* = 96)	6.2	0.8	5.9	5.7-6.3	5.1	10.8
Systolic BP (mmHg) (*n* = 110)	126	20.5	122	110-138	91	192
Diastolic BP (mmHg) (*n* = 110)	74	12	73	65-81	47	115
Fructosamine (μmol/L) (*n* = 86)	213	28.7	210	196-228.2	169	401

The total study population was 118, 8 patients has missing data for BP and 12 patients has missing data for BMI

^a^HbA1c was not reported in one participant due to the presence of variant Haemoglobin

^b^For the follow up OGTT we had data for 92 patients. Out of 118 who had baseline data, 14 were lost to follow up, 3 deceased, 8 did not have follow up OGTT because they were initiated on treatment for T2DM, and 1 did not have follow up OGTT due to HbA1c variant

### Prevalence of diabetes mellitus and impaired glycemia

#### Baseline assessments

##### Glycemic status based upon WHO criteria

At baseline, 48% (57/118) of participants had normal glucose tolerance (NGT), 32% (38/118) impaired glycaemic status (IGS) (which included 3% IFG (3/118), 25% IGT (30/118) and 4% IFG + IGT (5/118)) and 20% (23/118) met the criteria for T2DM.

Patients with T2DM were older and more obese (Table 2).

**Table 2:** Associations of means of basic parameters with background and 3 month glycaemic status

	NGT	IGS	T2DM	*P* value
Sex (Male)	47	30	19	0.89
Age (Years)	57+ 11	64+ 10	67+ 12	0.001
BMI (kg/m²)	27 + 4	29+ 4	31 + 7	0.02
Fructosamine	207 + 17	208+ 22	236 + 47	0.001
Ethnicity				
Afrocaribbean	3	2	0	0.75
Asian	9	5	5	
Caucasian	45	31	18	
Sex (Male)	41	22	17	0.38
Age (Years)	59+ 11	61+ 10	66+ 12	0.08
BMI (kg/m²)	28 + 5	28+ 5	32 + 5	0.008
Fructosamine	207 + 18	213+ 19	232 + 49	0.01
Ethnicity				
Afrocaribbean	2	1	1	0.55
Asian	7	7	3	
Caucasian	46	17	17	

### Glycaemic status based upon HBA1C criteria

At baseline, the prevalence of diabetes based upon an HbA1c >6.5% was 16%. Similar to the WHO criteria, patients with T2DM were older (age 60 + 11 and 67 + 11 for NGT vs. T2DM, *p* = 0.05) and more obese (BMI 28 + 5 vs. 31 + 6 for normal vs. T2DM, *p* = 0.04) compared to patients without T2DM.

### Glycaemic status: WHO vs. HBA1C (Figure 2a)

**Figure 2:**
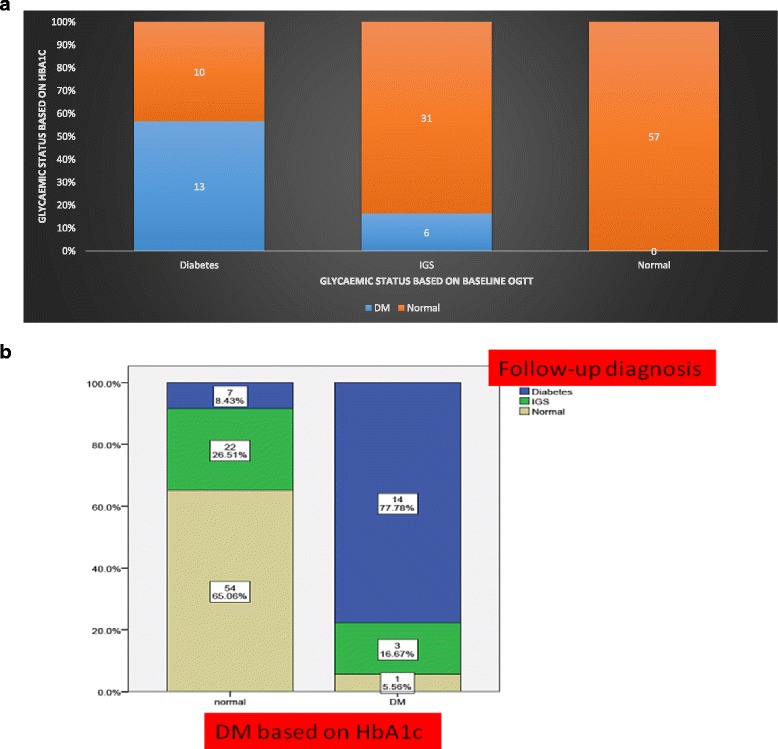
**a** Glycaemic status in the study population based on the WHO (x-axis) and HbA1c criteria (y-axis). **b** Glycaemic status in the study population based on the WHO criteria (Y axis) at 3 months and HbA1c (X axis) criteria at baseline

All patients classified as NGT on WHO criteria also had HbA1c ≤ 6.5% (48 mmol/mol) (Figure 2a). However 43% (*n* = 10/23) of the participants classified as having T2DM on the OGTT criteria would have been classified as patients without diabetes based on the HbA1c criteria. Out of 37 patients classified as IGS on the OGTT criteria 31 (84%) were classified as normal based on the HbA1c criteria.

### 3 month assessments

#### Glycaemic status at 3 months

The glycaemic status was reclassified at 3 months following discharge from hospital Data were available at this time point for 101 participants (14 drop outs, 3 died). All three participants who died had abnormal glycemia (2 IGT, 1 DM). At 3 months, 54% (55/101) of participants had NGT, 25% (25/101) IGS [9% (9/101) IFG, 11% (11/101) IGT and 5% (5/101) IFG + IGT)] and 21% (21/101) T2DM.

At the 3 months visit patients with T2DM were older and more obese than those with NGT (Table 2).

Comparing the glycaemic status based on the HbA1c and the OGTT criteria at 3 months showed that HBA1C would have incorrectly classified as normal 7 (33%) subjects with T2DM based on the 3 months OGTT (Figure 2b). Only one of the subjects classified as normal on the OGTT criteria at 3 months had T2DM based on the initial HbA1c (HbA1c criteria). Participants classified as having IGS at 3 months were mostly normal (88%) on the HbA1c criteria with only 3 (12%) having T2DM.

### Diabetes predictor score (DPS)

The screening algorithm had been designed to reduce the number of referrals for OGTT. We wanted to look at an algorithm which would perform well against WHO criteria as gold standard when used to detect T2DM. Therefore we examined clinical parameters that differed between patients with and without diabetes including age, BMI, sex, ethnicity, FPG, HbA1c, and nature of the cardiac event. Age, BMI, HbA1c, FPG differed between patients with and without diabetes and using logistic regression significant predictors of diabetes status were age, FPG and HbA1c (Table 3). The equation we designed based on the regression co-efficients was as follows:

**Table 3:** Logistic regression to identify statistically significant predictors of glycaemic status (diabetes vs. else) in order to compute the Diabetes Predictor Score

Variable	Regression co-efficient	Odds ratio	Confidence interval	*p* value
BMI	0.05	1.0	0.91-1.23	0.49
Age	0.1	1.1	1.03-1.19	0.007
HbA1c	1.6	4.8	1.19-19.1	0.03
FPG	1.7	5.4	1.89-15.8	0.002

Nagelkerke R^2^ 0.59

### Diabetes predictor score = (0.1 * Age) + (1.7 * FPG) + (1.6 * HbA1c)

**Figure 3:**
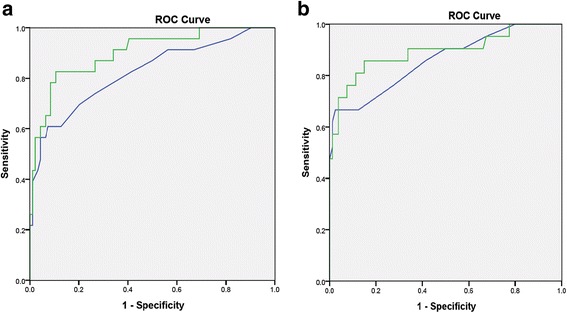
ROC curve analysis for the performance of the diabetes predictor score and HbA1c compared to WHO criteria to diagnose T2DM. A: at baseline, B: at 3 month

At baseline, compared to OGTT as the gold standard to detect T2DM the DPS had an AUC of 90% (*p* < 0.001) while A1C had an AUC of 82% (Figure 3). Using a cut-off at 26.32 achieved sensitivity of 83% with specificity of 87%. Positive and negative predictive values were 61 and 95% respectively. In comparison HBA1C criteria was associated with sensitivity of 57% and specificity of 94%. Positive and negative predictive values were 68% and 90% respectively.

At the 3 month visit, DPS had significant correlation with the WHO criteria, with an AUC of 89% (*p* < 0.001) while A1C had an AUC of 84% (Figure 3). Using a cut-off at 26.32 achieved sensitivity of 81 and specificity of 89%. Positive and negative predictive values were 66 and 95% respectively. In comparison HBA1C criteria was associated with sensitivity of 67% and specificity of 95%. Positive and negative predictive values were 78 and 91% respectively.

### The utility of the screening algorithm

By applying our screening algorithm, 20 out of 23 patients (87%) with T2DM would have been referred for an OGTT (i.e. 3 patients (13%) with T2DM based on OGTT would have been missed by the screening algorithm).

On the contrary, 42 out of 57 patients with normal OGTT results (74%) would not have been referred. In addition the screening algorithm would have detected 56% of the patients with IGT (These patients would have been referred for OGTT).

At 3 months, the use of the screening algorithm would have only missed 3 patients (14.3%) with T2DM. 18 out of 21 patients with DM would have been referred for an OGTT. In contrast, 36 out of 55 patients with normal OGTT results (65.5%) would not have been referred.

## Conclusions

In this study we have found that 51% of the patients admitted with ACS have either T2DM or IGS at baseline. However, two thirds of patients with T2DM or IGS at baseline changed their glycaemic status at 3 months. The HbA1C criteria would have miss-classified 43% of patients with T2DM at baseline on OGTT as NGT. Considering that OGTT may still be considered the “gold-standard” in this setting but is costly and time consuming, our screening algorithm managed to reduce the number of patients requiring OGTT significantly and only missed 13% of people with T2DM. Our diabetes predictor score had a good performance compared to OGTT-criteria at baseline and follow-up to diagnose T2DM.

The OGTT and HbA1c diagnostic criteria gave a different prevalence of diabetes in our cohort). By using the two different criteria we are identifying different cohort of patients with diabetes. Although the performance of the HbA1c criteria is better at 3 months, there are still differences in the classification of patients. The reason for the improved performance of the HbA1c criteria at 3 months may be due to HbA1c reflecting longer term glycemic status compared to OGTT and its better reproducibility. In addition to the clinical implications of inconsistencies in the diagnosis of T2DM in patients with ACS, there are important considerations with regards to the diagnosis of IGS. The detection of IGS has important implications for subsequent cardiovascular morbidity and mortality [10–15]. Patients with IGS are also known to progress to T2DM with studies suggesting an incidence of around 57.2 per 1000 patient years [27]. Other studies have suggested similar findings with incidence ranging from 35 to 58 per 1000 patient-years depending on ethnicity [28, 29]. IGT has also been known to be associated with cardiovascular mortality. Whitehall study suggested an increase in cardiovascular mortality by double in patients with abnormal OGTT results (2 h PG > 5.3 mmol/l) vs. normal Identification of IGT may afford opportunities for therapeutic life style modification or pharmacological interventions such as metformin which may slow the progression to T2DM [27] and impact cardiovascular outcomes In this report, all 3 participants who died had abnormal glycaemic status with two having IGS at baseline all of whom would have been classified as normal based upon the HbA1c criteria.

Our screening algorithm correlated well with the OGTT criteria. Overall, its use would lead to a reduction in the OGTTs. Moreover, an advantage compared to the use of an HbA1c measurements alone, use of the screening algorithm identifies at 50% of the participants with IGS. In comparison, the HbA1c criteria can detect less than 20% of IGS subjects and would label them all as having T2DM (Figure 2b).

The screening algorithm was originally designed to reduce the number of OGTTs required rather than to define glucose tolerance. We therefore evaluated a modified algorithm which performed better when compared to the OGTT criteria. Our DPS performed better than the HbA1c criteria in terms of sensitivity and NPV. However compared to the OGTT criteria, its positive predictive values were lower suggesting that it would classify some patients incorrectly as having T2DM therefore we propose that DPS is used to “exclude” diabetes.

Our study does have some limitations. The prevalence of diabetes in our study was lower compared to previous studies [10] which could impact some of our subsequent analysis. Our cohort of patients comprised 80% white Caucasians and so it is difficult to know whether these data apply equally to other ethnic groups. The follow up period of 3 months is relatively short and so may not predict longer term glycaemic status.

In patients with ACS, the use of differing diagnostic criteria has a significant impact on the glycaemic classification of patients which may have importance for longer term cardiovascular outcomes. Glycaemic classification during hospitalization, may not accurately predict the subsequent diabetes status particular in patients with IGS. The use of a screening algorithm and predictor score appears to be a better predictor of glycemic status compared to the HbA1c criteria when using OGTT criteria as a standard and may serve as useful tools for the assessment of hospitalized patients and reduce the number of OGTTs required. The utility of the screening algorithm and DPS in predicting glycaemic status and their impact on the need for OGTT need to be examined in larger cohorts with longer follow up duration (12-24 months).

## Abbreviations

ACS: Acute coronary syndrome
NSTEMI: Non-ST segment elevation myocardial infarction
STEMI: ST segment myocardial infarction
IGS: Impaired glycaemic status
T2DM: Type 2 Diabetes Mellitus
IGT: Impaired glucose tolerance
IFG: Impaired fasting glycaemia
OGTT: Oral glucose tolerance test
WHO: World Health Organization
DPS: Diabetes Predictor Score
NGT: Normal glucose tolerance
